# Radiomics predicts poorly differentiated hepatocellular carcinoma and uncovers ribosomal-immune dysregulation mechanism

**DOI:** 10.1016/j.isci.2026.114699

**Published:** 2026-01-19

**Authors:** Yiping Gao, Dong Liu, Yifan Miao, Zhiqian Lou, Ziwei Luo, Yonggang Li, Hongfa Cai, Yan Zhu, Shuangqing Chen

**Affiliations:** 1 Department of Radiology, The Affiliated Suzhou Hospital of Nanjing Medical University, Gusu School, Suzhou, Jiangsu 215000, China; 2 Department of Radiology, Third Affiliated Hospital of Naval Medical University, NO. 225 Changhai Road, Shanghai 200082, China; 3 Department of Radiology, First Affiliated Hospital of Soochow University, Suzhou, Jiangsu 215000, China

**Keywords:** cancer, molecular interaction, molecular network

## Abstract

Hepatocellular carcinoma (HCC) shows marked spatial heterogeneity, limiting biopsy-based Edmondson-Steiner (ES) grading. We developed a multicenter radiogenomic framework to noninvasively predict ES grade and explore underlying molecular mechanisms. Arterial-phase DCE-MRI from 295 patients and The Cancer Imaging Archive (TCIA) cases were analyzed using three tumor regions (body, edge, and out). An integrated volume-of-interest (VOI) random forest (RF) model was trained with selected features and externally validated. Radiogenomic analysis correlated radscore with TCIA transcriptomic profiles using weighted gene co-expression network analysis (WGCNA). The model achieved high discrimination (area under the curve [AUC] 0.959 internally; 0.860 externally). Radscore-associated modules revealed ribosomal dysregulation and immune exhaustion. A derived prognostic signature stratified and The Cancer Genome Atlas (TCGA) patients into distinct risk groups and independently predicted survival (hazard ratio [HR] 3.95, *p* < 0.0001; C index 0.643). This integrated radiogenomic approach enables noninvasive ES grading and provides insight into biologically relevant tumor heterogeneity.

## Highlights

• Integrated multi-VOI radiomics enables noninvasive Edmondson-Steiner grading in HCC
• An externally validated random forest model achieves robust multicenter performance
• Radiogenomic analysis links imaging heterogeneity to ribosomal dysregulation and immunity
• A radscore-derived signature independently predicts survival in TCGA-HCC patients

## Introduction

Primary liver cancer (PLC), comprising hepatocellular carcinoma (HCC, 85% of PLC), intrahepatic cholangiocarcinoma, and combined hepatocellular-cholangiocarcinoma,^1^ ranks as the fourth most common and the second deadliest malignancy globally.^2^

Pathological differentiation is a histological grading index for liver cancer that is tightly linked to prognosis in HCC.^3^ Poorly differentiated HCC exhibits frequent postoperative recurrence and microvascular invasion, often necessitating neoadjuvant *trans*-arterial chemoembolization or radiotherapy before resection. Conversely, well-differentiated tumors demonstrate indolent progression, where surgical resection or ablation may be given priority.^4^ Differentiation status further predicts systemic therapy response; poorly differentiated tumors may respond better to immune checkpoint inhibitors due to immunosuppressive microenvironments, while well-differentiated subtypes often rely on angiogenic pathways, and thus, benefit from anti-angiogenic agents.^5^^,^^6^ Consequently, preoperative differentiation assessment is of great value for precise treatment and risk stratification.

A growing number of studies have shown that HCC has a high degree of spatial heterogeneity,^7^ single-point biopsy only reflects the local state of the tumor and is difficult to capture the pathological differentiation heterogeneity of the entire tumor,^8^ particularly in poorly differentiated cases. This limitation underscores the need for non-invasive, whole-tumor evaluation methods.

Radiomics applied to dynamic contrast-enhanced magnetic resonance imaging (DCE-MRI) extracts quantitative features reflecting tumor heterogeneity and differentiation status via high-throughput feature extraction,^9^ enabling repeatable, dynamic monitoring. However, existing related studies suffer from critical deficiencies: single-center designs, small sample sizes, the absence of external validation, and superficial “image-to-prediction” approaches that lack mechanistic and multi-omics explanation.^10^^,^^11^^,^^12^

Peritumor regions, especially the tumor-liver interface (TLI) highlighted by Wu et al.,^13^ have shown promise for capturing more tumor microenvironment (TME) details.^14^^,^^15^ Yet, no prior study has comprehensively analyzed this high-yield target for HCC differentiation prediction.

To bridge these gaps, we developed and externally validated a multicenter DCE-MRI radiomics model for the non-invasive prediction of HCC differentiation (external area under the curve [AUC] = 0.87). We then integrated The Cancer Imaging Archive (TCIA), The Cancer Genome Atlas (TCGA) transcriptomic data via weighted gene co-expression network analysis (WGCNA), immune-infiltration analyses, and survival modeling to unravel the molecular pathways associated with our radiomic score. This combined radiogenomic approach not only yields a clinically practical predictive tool but also suggests that poorly differentiated HCC is linked to specific metabolic and immune-reprogramming pathways, offering novel insights for preoperative risk assessment and personalized therapy.

## Results

### Study workflow and patient characteristics

**Figure 1:**
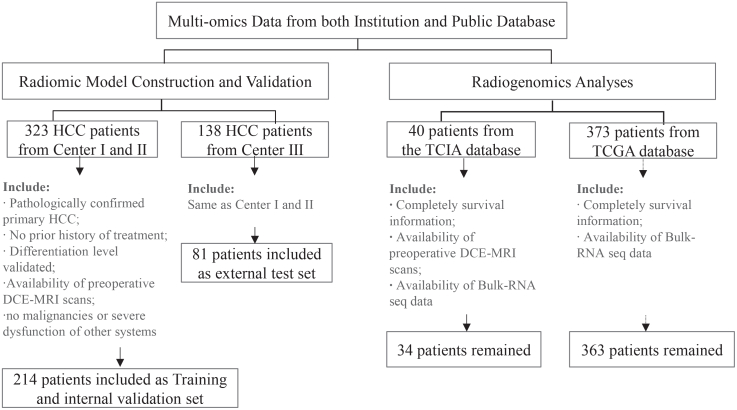
Study workflow and patient enrollment Flowchart illustrating patient selection from three clinical centers (Center I, *n* = 105; Center II, *n* = 109; Center III, *n* = 81) and public cohorts (The Cancer Imaging Archive [TCIA], *n* = 37; The Cancer Genome Atlas [TCGA], *n* = 363). Final cohorts were divided for model development (Centers I and II) and external validation (Center III and TCIA).

The enrollment and exclusion workflow is shown in Figure 1. The combined cohort comprised 295 institutional, 363 TCGA:TCGA.LIHC.sampleMap/HiSeqV2_PANCAN, and 34 TCIA:https://doi.org/10.7937/K9/TCIA.2016.IMMQW8UQ HCC patients. Demographic and clinical characteristics were comparable across centers (*p* > 0.05).

The mean age (±SD) was 58.2 ± 8.5 years in the cross-validation set, 57.8 ± 7.9 years in the hold-out test set, and 57 ± 7.5 years in the external test set, compared to 60 ± 13.8 years in TCGA and 58.5 ± 14.7 years in TCIA. Female patients comprised 22%–33% of each cohort. Median overall survival was 636 days in TCGA and 680 days in TCIA. Poorly differentiated tumors accounted for 56.7%–58.3% of institutional cases (Table 1).

**Table 1:** Baseline and grouping characteristics for patients in institution, TCGA and TCIA database

	Institution (*n* = 295)			TCGA	TCIA	*p* value
	Cross-validation set (*n* = 151)	Hold-out test set (*n* = 63)	External test set (*n* = 81)	(*n* = 363)	(*n* = 34)	
Age (Mean ± SD, years)	58.2 ± 8.5	57.8 ± 7.9	57 ± 7.5	60 ± 13.8	58.5 ± 14.7	0.218
Gender (female, %)	24.5%	23.2%	22.0%	31%	33%	0.327
Overall survival (medium, days)	/	/	/	636	680	0.287
Differentiation level (poor-differentiated, %)	58.3%	56.7%	57.5%	/	/	0.986

TCGA, The Cancer Genome Atlas; TCIA, The Cancer Imaging Archive; SD, standard deviation.

### Radiomics model performance

From the multi-region volume of interests (VOIs) across DCE-MRI sequences, 1,688 radiomic features were initially extracted and preprocessed. A total of 12 features remained in the integrated VOI, 31 in the edge-out VOI, 14 in the body-edge VOI, and 22 in the body-out VOI. Extracted feature lists and definitions are provided in Table S1.

**Figure 2:**
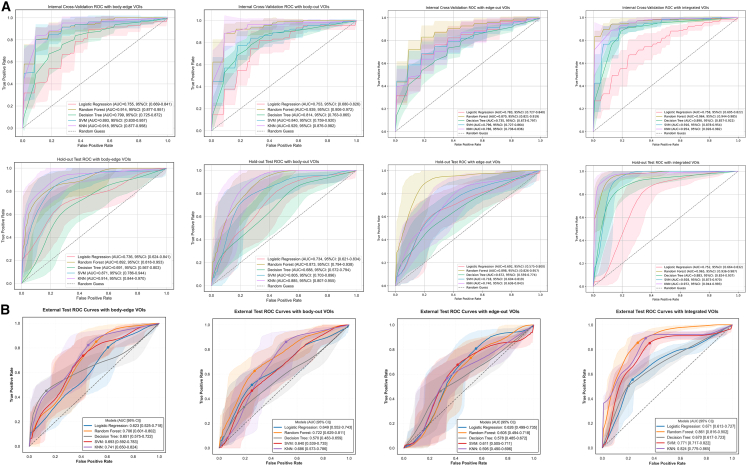
Radiomics model performance (A) Receiver operating characteristic (ROC) curves comparing different volumes of interests (VOIs) combinations during 10-fold cross-validation, including integrated VOIs, body-edge, edge-out, and body-out VOIs. (B) External validation performance across classifiers in the Center III cohort. Statistics in (A) and (B), area under the curve (AUC) values were estimated using bootstrap resampling (1,000 iterations) and are reported with 95% confidence intervals.

Table 2 presents performance metrics for 10-fold cross-validation and the hold-out test set. In cross-validation, the integrated VOI with k-nearest neighbor (KNN) and random forest (RF) models achieved the highest discrimination, with AUCs of 0.943 (95% CI, 0.915–0.970) and 0.959 (95% CI, 0.931–0.987), respectively. The integrated RF model yielded sensitivity = 0.922 (95% CI, 0.894–0.951), specificity = 0.836 (95% CI, 0.760–0.912), and F1 = 0.911 (95% CI, 0.889–0.932). Among paired VOI combinations, the KNN classifier on the body-edge VOI achieved an AUC of 0.958 (95% CI, 0.925–0.990), with sensitivity = 0.850 (95% CI, 0.765–0.935) and specificity = 0.864 (95% CI, 0.787–0.940). In the hold-out test set, the integrated RF maintained excellent accuracy (AUC = 0.986; 95% CI, 0.971–0.996; sensitivity = 0.960; specificity = 0.854; F1 = 0.936), outperforming all other models and VOIs. Overall, integrating all three VOIs consistently enhanced predictive performance (Figure 2A).

**Table 2:** Predicting results for differentiation level with different machine learning models in the cross-validation and internal test set

VOIs	10-fold cross-validation set				Hold-out test set			
	AUC (95% CI)	Sensitivity (95% CI)	Specificity (95% CI)	F1 score (95% CI)	AUC (95% CI)	Sensitivity (95% CI)	Specificity (95% CI)	F1 score (95% CI)
**Body-edge**								

RF	0.914 (0.893, 0.991)	0.839 (0.706, 0.972)	0.875 (0.809, 0.942)	0.900 (0.801, 0.999)	0.892 (0.818, 0.953)	0.702 (0.543, 0.844)	0.702 (0.543, 0.844)	0.719 (0.603, 0.827)
LR	0.755 (0.669, 0.841)	0.782 (0.658, 0.906)	0.620 (0.482, 0.758)	0.735 (0.624, 0.847)	0. 736 (0.624, 0.841)	0.734 (0.588, 0.867)	0.583 (0.451, 0.708)	0.637 (0.506, 0.745)
SVM	0.893 (0.830, 0.957)	0.804 (0.672, 0.936)	0.818 (0.748, 0.888)	0.882 (0.785, 0.979)	0.871 (0.786, 0.944)	0.837 (0.718, 0.943)	0.856 (0.750, 0.943)	0.826 (0.732, 0.913)
KNN	0.918 (0.877, 0.958)	0.850 (0.765, 0.935)	0.864 (0.787, 0.940)	0.839 (0.780, 0.897)	0.914 (0.844, 970)	0.837 (0.706, 0.947)	0.812 (0.690, 0.917)	0.803 (0.694, 0.896)
DT	0.799 (0.725, 0872)	0.735 (0.588, 0.881)	0.761 (0.678, 0.844)	0.716 (0.598, 0.833)	0.691 (0.567, 0.803)	0.484 (0.316, 0.647)	0.646 (0.509, 0.780)	0.495 (0.351, 0.627)

**Edge-out**								

RF	0.870 (0.821, 0.919)	0.808 (0.723, 0.892)	0.827 (0.733, 0.922)	0.804 (0.764, 0.844)	0.898 (0.826, 0.957)	0.879 (0.767, 0.972)	0.746 (0.617, 0.872)	0.809 (0.713, 0.889)
LR	0.783 (0.727, 0.840)	0.710 (0.620, 0.800)	0.755 (0.668, 0.842)	0.713 (0.627, 0.799)	0.692 (0.575, 0.800)	0.715 (0.575, 0.842)	0.561 (0.419, 0.705)	0.640 (0.518, 0.748)
SVM	0.796 (0.727, 0.864)	0.629 (0.557, 0.701)	0.755 (0.644, 0.865)	0.660 (0.587, 0.734)	0.718 (0.604, 0.819)	0.736 (0.590, 0.872)	0.669 (0.540, 0.800)	0.696 (0.571, 0.800)
KNN	0.786 (0.736, 0.836)	0.688 (0.571, 0.805)	0.627 (0.524, 0.731)	0.648 (0.556, 0.739)	0.740 (0.636, 0.843)	0.785 (0.660, 0.906)	0.603 (0.447, 0.745)	0.700 (0.589, 0.800)
DT	0.735 (0.673, 0.797)	0.704 (0.603, 0.806)	0.727 (0.587, 0.868)	0.698 (0.627, 0.770)	0.672 (0.559, 0.774)	0.641 (0.488, 0.786)	0.664 (0.532, 0.790)	0.630 (0.500, 0.750)

**Body-out**								

RF	0.939 (0.906, 0.972)	0.878 (0.799, 0.957)	0.915 (0.821, 1.000)	0.882 (0.828, 0.936)	0.872 (0.792, 0.938)	0.908 (0.811, 0.979)	0.730 (0.595, 0.854)	0.834 (0.747, 0.909)
LR	0.753 (0.680, 0.826)	0.565 (0.430, 0.701)	0.723 (0.599, 0.846)	0.575 (0.485, 0.664)	0.733 (0.629, 0.830)	0.750 (0.620, 0.870)	0.637 (0.500, 0.766)	0.709 (0.604, 0.817)
SVM	0.840 (0.759, 0.920)	0.778 (0.659, 0.896)	0.757 (0.619, 0.895)	0.745 (0.644, 0.846)	0.806 (0.709, 0.890)	0.770 (0.643, 0.891)	0.752 (0.630, 0.875)	0.762 (0.653, 0.854)
KNN	0.929 (0.876, 0.982)	0.935 (0.910, 0.961)	0.880 (0.770, 0.989)	0.871 (0.812, 0.929)	0.885 (0.799, 0.953)	0.885 (0.789, 0.962)	0.729 (0.590, 0.865)	0.819 (0.723, 0.894)
DT	0.814 (0.763, 0.865)	0.722 (0.597, 0.848)	0.843 (0.753, 0.933)	0.743 (0.677, 0.808)	0.688 (0.578, 0.784)	0.589 (0.426, 0.737)	0.612 (0.469, 0.763)	0.597 (0.466, 0.714)

**Integrated**								

RF	0.959 (0.931, 0.987)	0.922 (0.894, 0.951)	0.836 (0.760, 0.912)	0.911 (0.889, 0.932)	0.986 (0.971, 0.996)	0.960 (0.917, 0.991)	0.854 (0.754, 0.936)	0.936 (0.896, 0.968)
LR	0.649 (0.587, 0.712)	0.590 (0.520, 0.660)	0.643 (0.556, 0.730)	0.647 (0.602, 0.693)	0.654 (0.558, 0.739)	0.661 (0.566, 0.755)	0.553 (0.431, 0.678)	0.679 (0.602, 0.749)
SVM	0.870 (0.826, 0.915)	0.836 (0.795, 0.877)	0.793 (0.698, 0.889)	0.851 (0.837, 0.865)	0.885 (0.821, 0.934)	0.817 (0.735, 0.891)	0.839 (0.743, 0.925)	0.853 (0.796, 0.904)
KNN	0.943 (0.915, 0.970)	0.900 (0.833, 0.968)	0.842 (0.787, 0.898)	0.898 (0.856, 0.941)	0.981 (0.961, 0.994)	0.930 (0.875, 0.970)	0.872 (0.783, 0.951)	0.925 (0.886, 0.962)
DT	0.881 (0.840, 0.923)	0.870 (0.819, 0.921)	0.862 (0.798, 0.927)	0.889 (0.854, 0.924)	0.897 (0.843, 0.944)	0.809 (0.720, 0.882)	0.824 (0.730, 0.919)	0.843 (0.787, 0.891)

VOIs, volumes of interest; AUC, area under curves; 95% CI, 95% confidence interval; RF, random forest; LR, logistic regression: SVM, support vector machine; KNN, k-nearest neighbor; DT, decision tree.

Next, we applied each classifier to the independent external cohort from the Center III (Figure 2B and Table 3). For the integrated VOI, RF again showed superior performance (AUC = 0.860; 95% CI, 0.818–0.899), with sensitivity = 0.707 (95% CI, 0.655–0.756), specificity = 0.847 (95% CI, 0.784–0.908), and F1 = 0.800 (95% CI, 0.762–0.834). KNN on the integrated VOI also performed well (AUC = 0.825; 95% CI, 0.780–0.865; sensitivity = 0.930; specificity = 0.872; F1 = 0.925). Models based on paired VOI combinations showed moderate discrimination: KNN on the body-edge VOI had AUC = 0.741 (95% CI, 0.641–0.834), while edge-out and body-out VOIs yielded lower AUCs (0.648 and 0.763, respectively).

**Table 3:** Predicting effect for differentiation level with different machine learning methods in the external test set

	RF (95% CI)	LR (95% CI)	SVM (95% CI)	KNN (95% CI)	DT (95% CI)
**Body-edge**					

AUC	0.706 (0.601, 0.802)	0.623 (0.525, 0.718)	0.693 (0.592, 0.783)	0.741 (0.641, 0.834)	0.651 (0.575, 0.783)
Sensitivity	0.442 (0.372, 0.514)	0.591 (0.519, 0.663)	0.464 (0.389, 0.540)	0.590 (0.519, 0.661)	0.468 (0.394, 0.538)
Specificity	08354 (0.735, 0.952)	0.611 (0.452, 0.762)	0.810 (0.675, 0.927)	0.711 (0.564, 0.851)	0.607 (0.447, 0.758)
F1 score	0.599 (0.529, 0.667)	0.704 (0.644, 0.761)	0.615 (0.543, 0.684)	0.713 (0.653, 0.767)	0.602 (0.528, 0.665)

**Body_out**					

AUC	0.721 (0.629, 0.810)	0.651 (0.561, 0.737)	0.638 (0.537, 0.736)	0.684 (0.574, 0.780)	0.572 (0.480, 0.660)
Sensitivity	0.525 (0.451, 0.598)	0.700 (0.556, 0.836)	0.590 (0.518, 0.661)	0.568 (0.497, 0.637)	0.541 (0.463, 0.612)
Specificity	0.833 (0.694, 0.729)	0.700 (0.642, 0.756)	0.573 (0.415, 0.730)	0.698 (0.548, 0.838)	0.551 (0.393, 0.717)
F1 score	0.354 (0.256, 0.452)	0.700 (0.642, 0.756)	0.700 (0.640, 0.756)	0.694 (0.635, 0.750)	0.659 (0.591, 0.717)

**Edge-out**					

AUC	0.605 (0.494, 0.718)	0.626 (0.499, 0.735)	0.611 (0.505, 0.711)	0.595 (0.490, 0.696)	0.578 (0.485, 0.672)
Sensitivity	0.405 (0.335, 0.478)	0.365 (0.294, 0.435)	0.405 (0.337, 0.480)	0.348 (0.279, 0.417)	0.528 (0.459, 0.601)
Specificity	0.796 (0.658, 0.915)	0.652 (0.488, 0.800)	0.652 (0.500, 0.800)	0.801 (0.667, 0.914)	0.666 (0.602, 0.728)
F1 score	0.558 (0.486, 0.631)	0.505 (0.430, 0.577)	0.546 (0.475, 0.617)	0.499 (0.420, 0.575)	0.437 (0.339, 0.535)

**Integrated**					

AUC	0.860 (0.818, 0.899)	0.671 (0.611, 0.727)	0.772 (0.718, 0.819)	0.825 (0.780, 0.865)	0.670 (0.618, 0.718)
Sensitivity	0.707 (0.655, 0.756)	0.696 (0.646, 0.744)	0.651 (0.596, 0.705)	0.621 (0.569, 0.671)	0.525 (0.471, 0.581)
Specificity	0.847 (0.784, 0.908)	0.591 (0.500, 0.680)	0.744 (0.664, 0.821)	0.856 (0.791, 0.915)	0.752 (0.672, 0.824)
F1 score	0.800 (0.762, 0.834)	0.751 (0.709, 0.789)	0.744 (0.700, 0.784)	0.741 (0.699, 0.780)	0.648 (0.601, 0.696)

**Figure 3:**
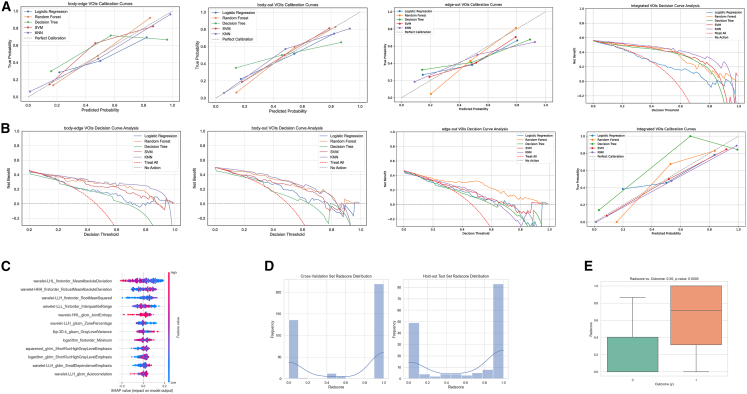
Model interpretation and radscore validation (A) Decision curve analysis showing net benefit of radiomics models versus “treat all” and “no intervention” strategies across threshold probabilities. (B) Calibration curves of machine learning classifiers evaluating agreement between predicted and observed differentiation. (C) SHapley Additive exPlanations (SHAP) summary plot highlighting top radiomic features influencing differentiation prediction. (D) Distribution of radscores in cross-validation and hold-out test sets. (E) Spearman correlation between radscore and Edmondson-Steiner grading (R = 0.500, *p* < 0.001). Statistics in (E), Spearman correlation, two-sided test.

The decision curve analysis curve illustrated the clinical utility of each VOI-based model versus “treat all” and “treat none” strategies, showing that both KNN and RF on the integrated VOI had the highest net benefit across threshold probabilities of 0.2–0.8, indicating that using the integrated radiomics could better identify low-differentiation tumors while minimizing unnecessary interventions (Figure 3A).

Calibration curves (Figure 3B) revealed that support vector machineand KNN on the integrated VOI exhibited the most stable agreement between predicted and observed differentiation status, closely following the 45° ideal line across the risk spectrum. RF and decision tree models showed good overall calibration, with modest overestimation at the highest predicted risks.

SHapley Additive exPlanations (SHAP) analysis of the integrated VOI highlighted four most influential features: “wavelet-LHL_firstorder_MeanAbsoluteDeviation,” “wavelet-HHH_firstorder_RobustMeanAbsoluteDeviation,” “wavelet-LLH_firstorder_RootMeanSquared,” and “wavelet-LLL_firstorder_InterquartileRange” (Figure 3C). Figure 3D shows the radscore distribution in the cross-validation and hold-out test sets. Figure 3E confirms its strong correlation with differentiation level (R = 0.500, *p* < 0.001).

### Radiogenomic analyses

**Figure 4:**
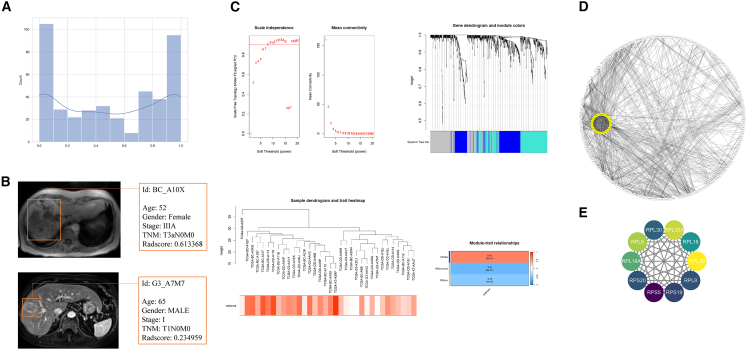
Radiogenomic associations and hub gene identification (A) Workflow of radscore computation in the TCIA cohort. (B) Representative DCE-MRI images, low-radscore HCC (uniform enhancement, pseudocapsule) versus high-radscore HCC (necrosis, irregular margins). (C) Weighted gene co-expression network analysis (WGCNA) module-trait relationships showing correlation between gene modules and radscore. (D) Protein-protein interaction (PPI) network of hub genes associated with radscore (MCODE score >3). (E) Hub genes identified using CytoHubba algorithms (degree >20, MCC top 10). Correlation coefficients and *p* values are shown in (C). PPI networks were generated using STRING.

We calculated the radscore for TCIA patients using the integrated RF model (Figure 4A). Representative cases (Figure 4B) show that low-radscore tumors exhibit uniform density, clear boundaries, pseudocapsules, and mild enhancement, whereas high-radscore tumors are irregular with necrosis and heterogeneous enhancement. Lower radscore correlated with lower BCLC and TNM stages, consistent with a better prognosis in non-poor differentiation.

WGCNA with a soft threshold of β = 10 identified three gene modules from radscore-correlated genes (Figure 4C). Turquoise and blue modules were tightly associated with radscore (cor = 0.34, *p* < 0.05). STRING network analysis showed turquoise-module genes enriched in ribosomal biosynthesis and translation, while blue-module genes correlated with immune response. Cytoscape MCODE/CytoHubba identified ten hub genes (Figures 4D and 4E): RPL30 (ribosomal protein L30), RPL35A (ribosomal protein L35a), RPL6 (ribosomal protein L6), RPL18A (ribosomal protein L18A), RPS20 (ribosomal protein S20), RPS5 (ribosomal protein S5), RPS19 (ribosomal protein S19), RPL18 (ribosomal protein L18), RPL29 (ribosomal protein L29), and RPL8 (ribosomal protein L8). Consistent with their proposed role in tumorigenesis, all ten hub genes were significantly upregulated in HCC tumors compared to normal tissues in the TCGA-LIHC cohort (log2FC > 0, *p* < 0.05; Figure S1).

### Immune-related and mechanism analyses

**Figure 5:**
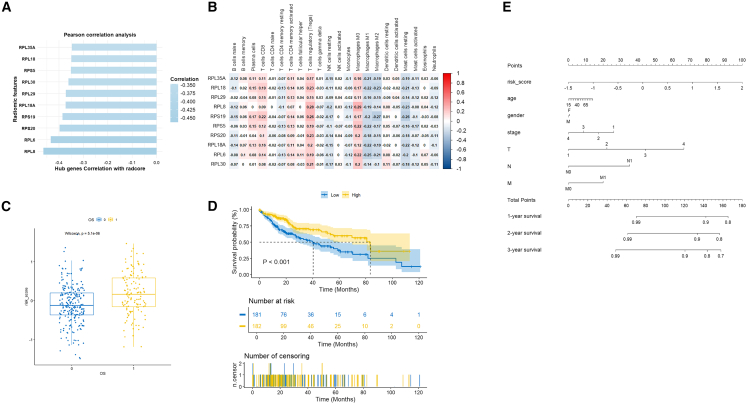
Prognostic risk model based on radiogenomic hub genes (A) Spearman correlations between hub gene expression and radscore. (B) Immune cell infiltration profiles associated with radscore via CIBERSORT analysis. (C) The patients in death status had the higher risk score compared to the patients in surive. (D) Kaplan-Meier survival curves comparing high-versus low-risk groups in the TCGA-LIHC cohort (HR = 3.95, *p* < 0.0001). (E) Nomogram integrating the risk score and clinical features for overall survival prediction. Statistics in (A), correlation assessed using two-sided tests. Statistics in (C) statistical significance assessed using the log rank test with a two-sided adjusted *p* < 0.05.

All hub genes correlated negatively with radscore (Figure 5A). CIBERSORT analysis (Figure 5B) demonstrated radscore-immunome interactions that ribosomal genes showed positive correlations with naive B cells (r = 0.12–0.15), negative correlations with monocytes (r ≤ −0.17), and varied associations with T cell subsets; notably, RPL8 had opposing effects on naive vs. memory T cells (r = +0.28 vs. −0.23, *p* < 0.001), suggesting ribosomal dysregulation impairs T cell differentiation in poorly differentiated HCC.

A multivariable Cox regression based on hub genes stratified patients into high- and low-risk groups (C index = 0.591, *p* < 0.001; Figure 5C). High-risk patients had significantly worse OS than low-risk patients (*p* < 0.001; Figure 5D). A nomogram validated by Harrell’s C index and 1,000 bootstrap resamples (C index = 0.643) confirmed the gene-based risk score as an independent mortality predictor (HR = 3.95, *p* < 0.0001; Figure 5E).

### Functional enrichment and tumor microenvironment

**Figure 6:**
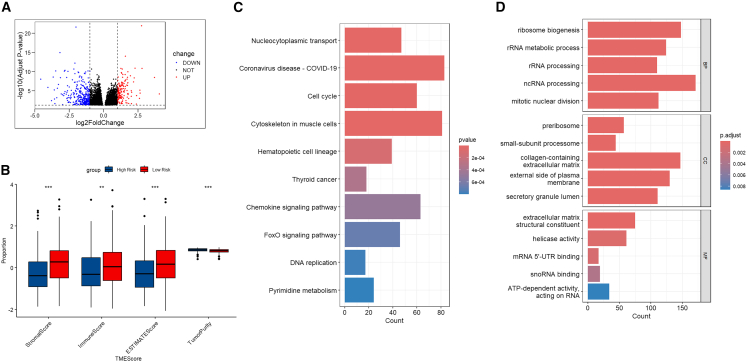
Functional enrichment and tumor microenvironment analysis (A) Volcano plot of differentially expressed genes (DEGs) between high- and low-risk groups. (B) Comparison of ESTIMATE scores-tumor purity, stromal score, and immune infiltration—between risk groups. (C) Kyoto Encyclopedia of Genes and Genomes (KEGG) pathway enrichment of DEGs, highlighting involvement in nucleocytoplasmic transport and FoxO signaling. (D) Gene ontology (GO) enrichment analysis demonstrating biological processes and molecular functions related to ribosomal dysregulation. Statistics in (A), DEGs were defined by |log2 fold change| > 2 and two-sided adjusted *p* < 0.05.

Differentially expressed genes (DEGs) between risk groups (|log_2_ FC| > 2, *p* < 0.05) were identified (Figure 6A). High-risk patients had significantly higher ImmuneScore, StromalScore, and ESTIMATE score, and lower tumor purity compared to low-risk patients (Figure 6B), supporting the validity of WGCNA modules.

KEGG pathway analysis revealed enrichment in nucleocytoplasmic transport, cell cycle regulation, FoxO signaling, and chemokine signaling (Figure 6C). GO analysis showed DEGs enriched in ribosomal biogenesis, rRNA processing, and mitotic nuclear division; cellular components localized to pre-ribosomal complexes; and molecular functions included extracellular matrix structural constituent and RNA binding (Figure 6D). These processes reflect accelerated protein synthesis machinery in poorly differentiated HCC.

## Discussion

HCC displays marked spatial heterogeneity, complicating clinical management. This multicenter study establishes a non-invasive radiogenomic framework that reliably predicts poorly differentiated HCC by integrating features from the TLI. Furthermore, we uncover a previously undescribed association between ribosomal dysregulation and local immune exhaustion, positioning the radscore as a dual-purpose biomarker for diagnosis and therapy selection.

Previous radiomic studies have been constrained by single-center designs and a narrow focus on the tumor core, limiting generalizability and biological insight.^16^^,^^17^ In this study, we developed a multi-VOI radiomics framework based on arterial-phase DCE-MRI. The RF model based on integrated VOIs achieved exceptional performance in both cross-validation (AUC = 0.959) and external validation (AUC = 0.860), outperforming alternative models and region combinations. The superior predictive performance reinforces the value of peritumoral regions as a rich source of biomarkers, corroborating emerging biological evidence of the invasive zone’s role in immunosuppression and progression. To further clarify the contribution of each spatial compartment, we performed supplementary single-VOI ablation experiments (Figure S2). These analyses were restricted to internal cross-validation and a hold-out test set, as their stand-alone performance was substantially lower than the integrated model, which is consistent with the known complementary nature of intra-tumoral, margin, and peritumoral phenotypes. Meanwhile, given that boosting and the random forest often register similar performance outcomes but bring different results in permutation importance and SHAP plots. We performed a head-to-head comparison between the RF and the XGBoost, including cross-validated and hold-out test performance metrics, permutation importance side-by-side, SHAP summary plots for each model, and Gini importance for RF model (Figure S3). While both models achieve comparable performance (AUC ∼0.86–0.90), RF offers more stable importance rankings, whereas XGBoost provides finer granularity in directionality at the cost of higher variance. This is mainly because that permutation importance quantifies the drop in model performance when a feature is shuffled, directly measuring predictive strength, while SHAP plots complement impurity/permutation methods by providing intuitive visualization of feature effects. The choice of model depends on the trade-off between interpretability (RF) and precision (XGBoost).

In recent years, multimodal artificial intelligence has garnered significant attention in medical imaging and oncology, aiming to integrate diverse data forms such as genomic, radiomic, and clinical features to improve predictive performance and biological insight.^18^^,^^19^ While the present study does not incorporate all potential data modalities, our multi-region radiomic approach combined with transcriptomic profiling represents a step toward multimodal integration. By capturing tumor heterogeneity through multiple volumes of interest and linking imaging features to molecular pathways, we demonstrate the potential of combining image-based and genetic data. Future work could further enhance this framework by incorporating additional modalities, such as histopathological whole-slide images, proteomic data, or clinical variables, to build more comprehensive and robust predictive models.

The integrated VOIs captured TME heterogeneity that was overlooked by single-region approaches, and notably, the incorporation of the TLI region emerged as a critical biomarker source. ComBat batch correction was employed to ensure generalizability across institutions.^20^ Overall, the radscore not only predicted tumor differentiation status but also served as a surrogate for ribosomal-immune dysregulation, offering a dual diagnostic-prognostic utility.

WGCNA identified a ribosomal gene module whose hub genes were significantly correlated with radscore. Functional enrichment revealed that high-radscore tumors—classified as poorly differentiated (ES grade III/IV) by our model—exhibited upregulation of ribosomal biogenesis and dysregulated nucleocytoplasmic transport. The established roles of ribosomal proteins in fundamental processes such as DNA replication, transcription, repair, and apoptosis position their dysregulation as a direct driver of dedifferentiation in HCC.^21^ The tight relationship of ribosomal proteins with cell development and differentiation through their extra-ribosomal functions means that their aberrant expression can disrupt transcriptional and epigenetic programs, ultimately promoting a less differentiated, malignant state.^22^ This is evidenced in HCC by RPL35A downregulation suppresses HCC cell proliferation,^23^ RPL30 acting as a core epigenetic factor in hepatocarcinogenesis,^24^ and RPL6^25^/RPS5^26^ stabilizing HIF-1α to promote angiogenesis and metastasis. These alterations accelerate oncoprotein synthesis and promote cell cycle progression, providing a mechanistic explanation for the aggressive behavior of poorly differentiated HCC. Specifically, ribosomal hyperactivity may drive proteomic instability, contributing to dedifferentiation and therapy resistance—a molecular basis for the poor prognosis observed in high-risk patients.

CIBERSORT analysis of radscore-associated hub genes revealed significant interactions with the immune microenvironment. Naive B cell infiltration positively correlated with radscore (r = 0.12–0.28), while monocytes were inversely correlated (r ≤ −0.17), suggesting compensatory humoral activation alongside impaired antigen presentation. Notably, RPL8 exhibited dichotomous associations with T cell subsets—positive with naive T cells (r = +0.28) but negative with memory T cells (r = −0.23, *p* < 0.001)—indicating ribosomal-driven T cell exhaustion. These patterns are consistent with the immunosuppressive microenvironment typically seen in poorly differentiated HCC.^27^^,^^28^

These findings have immediate translational implications. The radscore could serve as a preoperative biomarker for stratifying patients for adjuvant therapies: high-radscore tumors, identified as poorly differentiated by our model, may benefit from ribosomal-targeted agents or immune checkpoint inhibitors, whereas low-radscore tumors may be appropriately managed with standard resection and anti-angiogenic therapy.

In a word, we present a validated radiogenomic framework that noninvasively predicts HCC differentiation and uncovers its mechanistic basis in ribosomal-immune dysregulation. The radscore serves both as a diagnostic biomarker and a therapeutic compass, guiding precision surgery planning, immunotherapy, and ribosomal inhibitor selection, and dynamic treatment response monitoring. This work bridges radiology, genomics, and immunology, advancing toward personalized HCC management.

### Limitations of the study

Several limitations should be acknowledged. First, reliance on arterial-phase MRI alone may overlook complementary biological information available from other sequences; future work will integrate multiparametric and functional imaging data. Second, despite multicentricity, the retrospective design necessitates validation in a prospective trial. Third, the proposed mechanisms require experimental confirmation. Third, the slight decrease in AUC from internal cross-validation (0.959) to external validation (0.860) can be attributed to several factors. First of all, the external cohort was acquired from a different institution with variations in MRI scanners, imaging protocols, and contrast agents. Meanwhile, patient population differences may introduce heterogeneity. Nevertheless, the external AUC of 0.860 remains clinically relevant and demonstrates the model’s generalizability within an acceptable range (less than 10%). Finally, correlating the radscore with actual response to immune checkpoint inhibitors in dedicated cohorts will be critical to confirm its predictive value.

## Resource availability

### Lead contact

Requests for further information and resources should be directed to and will be fulfilled by the lead contact, Shuangqing Chen (sznaonao@163.com).

### Materials availability

This study did not generate new unique reagents.

### Data and code availability

• The public datasets used and/or analyzed during the current study are available from the databases of TCGA (https://portal.gdc.cancer.gov/) and TCIA (https://www.cancerimagingarchive.net/). Any additional information required is available upon reasonable request to the lead contact.
• The code for radiomic feature extraction and model training is available at (https://github.com/YPGao915/radiomics-ml-pipeline.git).

## Acknowledgments

Appreciation to the TCIA and TCGA databases for providing public data.

## Author contributions

Conception and design, Y.G. and S.C.; methodology, Y.G.; resources, Y.G., D.L., Y.M., Zhiqian Lou, Ziwei Luo, Y.L., H.C., and Y.Z.; writing – original draft, Y.G.; writing – review and editing, Y.G. and S.C.; supervision, S.C.

## Declaration of interests

All authors have no conflicts of interest.

## STAR★Methods

### Key resources table

**Table:** 

REAGENT or RESOURCE	SOURCE	IDENTIFIER
**Biological samples**		

TCGA-LIHC	National Cancer Institute	https://tcga-xena-hub.s3.us-east-1.amazonaws.com/download/TCGA.LIHC.sampleMap%2FHiSeqV2_PANCAN.gz
TCIA-LIHC	National Cancer Institute	https://doi.org/10.7937/K9/TCIA.2016.IMMQW8UQ

**Deposited data**		

GitHub	Code for radiomic feature extraction and model training	https://github.com/YPGao915/radiomics-ml-pipeline.git

**Software and algorithms**		

3D Slicer v5.2.0	Brigham and Women’s Hospital, Harvard Medical School	https://www.slicer.org/
Cytoscape	Cytoscape Consortium	https://cytoscape.org/
STRING	The Swiss Institute of Bioinformatics and ETH Zurich	https://string-db.org/
Python v3.7/3.11	Python Software Foundation	https://www.python.org/
R studio v4.3.3	R Foundation	https://cran.r-project.org/

### Experimental model and study participant details

#### Human participants

This study included human HCC patients from both retrospective institutional cohorts and publicly available datasets. This study was approved by the local Institutional Review Boards (IRBs) of the participating centers, with a waiver of informed consent due to the retrospective nature of the study and the use of anonymized data, and ethical approval information for public datasets was provided by the original data contributors and governing institutions.

For the institutional cohorts, patients were retrospectively identified from three independent medical centers (Center I, Center II, and Center III). Eligibility criteria included adults (>18 years) with histopathologically confirmed primary HCC who underwent pre-treatment DCE-MRI. Patients with prior liver surgery, unclear imaging data, extrahepatic malignancies, or severe hepatic, renal, or cardiac dysfunction were excluded. Publicly available human imaging and transcriptomic datasets were obtained from TCIA and TCGA-LIHC cohort. All public datasets were de-identified and accessed in accordance with their respective data usage policies.

Demographic variables, including age and gender information was available for the majority of patients in the institutional cohorts and public datasets and were included as descriptive variables, the results showed no significant statistical differences among various cohorts. Variables not reported in the source datasets were recorded as not available.

The total sample size for each cohort was defined *a priori* based on data availability. Patients were allocated to training, internal validation, and external validation cohorts according to institutional source, reflecting the retrospective and multicenter study design.

### Method details

#### Study design and patient enrollment

This retrospective, multi-center study enrolled patients with histopathologically confirmed HCC from three independent institutions. All included patients underwent pre-treatment DCE-MRI and had documented tumor differentiation status.

Inclusion criteria were: (1) age >18 years; (2) pathologically confirmed primary HCC; (3) preoperative MRI performed within 30 days before surgery; (4) absence of extrahepatic malignancies or severe hepatic, renal, or cardiac dysfunction; and (5) available histopathological differentiation grading. Exclusion criteria included: (1) inadequate or incomplete DCE-MRI data; and (2) history of prior liver surgery.

Patients from Centers I and II were randomly split into a training cohort and an internal validation cohort at a 7:3 ratio. Patients from Center III and TCIA constituted independent external validation cohorts.

In parallel, publicly available datasets were incorporated for radiogenomic analyses, including 37 DCE-MRI cases from TCIA (https://doi.org/10.7937/K9/TCIA.2016.IMMQW8UQ) and 363 RNA-sequencing samples with matched clinical annotations from TCGA-LIHC project (https://portal.gdc.cancer.gov/).

#### Histopathological evaluation

Tumor differentiation was assessed according to the ES grading system. ES grades I and II were categorized as non-poor differentiation, whereas ES grades III and IV were classified as poor differentiation. All pathological diagnoses were established by experienced hepatopathologists at each participating center.

#### MRI acquisition and preprocessing

All MRI examinations were performed using either 1.5 T or 3 T scanners following standardized liver imaging protocols at each institution. Imaging sequences included pre-contrast T1-weighted images and arterial-phase images after intravenous gadolinium administration. Detailed scanner models and acquisition parameters are provided in File S1. DICOM images were retrieved from local PACS and anonymized prior to analysis.

#### Tumor segmentation and VOI definition

Three-dimensional VOI segmentation was performed on the dominant contrast-enhanced phase using 3D Slicer software (version 5.2.0). Two board-certified radiologists independently delineated tumor boundaries while excluding intratumoral vessels and the tumor capsule. Discrepancies were resolved by consensus under the supervision of a senior radiologist.

Three VOIs were defined for each tumor:

Body-VOI: the entire tumor volume;
Edge-VOI: a 3-mm ring spanning the TLI in both inward and outward directions;
Out-VOI: a 3-mm peritumoral extension outside the TLI.

Pairwise and integrated VOI combinations (Body-Edge, Body-Out, Edge-Out, and Body-Edge-Out) were constructed to evaluate complementary spatial information. The 3-mm thickness was selected based on prior studies demonstrating that peritumoral regions within 3–5 mm capture invasive behavior and microenvironmental alterations relevant to tumor aggressiveness.

#### Radiomic feature extraction and harmonization

All images were resampled to an isotropic voxel size of 3 × 3 × 3 mm^3^ and *Z* score normalized. Radiomic feature extraction was conducted using PyRadiomics (https://pyradiomics.readthedocs.io/), generating first-order statistics, shape features, and texture features.

To minimize scanner-related batch effects across centers, ComBat harmonization was applied after feature extraction. The harmonization protocol is detailed in File S1.

#### Feature selection strategy

Feature selection was performed exclusively in the training cohort to prevent data leakage. Outliers were first removed using the IQR method. Highly correlated feature pairs were identified using Spearman correlation analysis (|ρ| > 0.75, *p* < 0.05); within each pair, the feature exhibiting stronger correlation with tumor differentiation was retained.

Subsequently, LASSO regression was applied, with the regularization parameter λ selected by minimizing binomial deviance. To ensure feature robustness across spatial regions, stability selection was performed. Features were retained if they appeared in more than 60% of two-VOI combinations or more than 90% of integrated VOIs. The stricter threshold for multi-VOI models was chosen to account for increased variability arising from segmentation uncertainty and boundary effects.

#### Model development and validation

Five classifiers—LR, RF, SVM, KNN, and DT—were trained using the selected features. Hyperparameters were optimized using 10-fold cross-validation with SMOTE applied to address class imbalance. The optimized model was applied unchanged to the external validation cohorts.

The radscore was uniformly defined as the predicted probability of poor differentiation (ES III/IV). For linear models, linear predictors were transformed using the logistic function to ensure consistency with probability outputs from non-linear classifiers.

#### Radiogenomic analysis

Radscore values were computed for TCIA patients using the integrated random forest model. Spearman correlation analysis (|R| > 0.3, *p* < 0.05) was used to identify radscore-associated genes. These genes were subsequently analyzed in the TCGA-LIHC cohort using WGCNA.

Outlier samples were excluded by hierarchical clustering. A soft-thresholding power achieving scale-free topology (R^2^ > 0.9) was selected. Co-expression modules were identified using dynamic tree cutting (minModuleSize = 30; MEDissThres = 0.25), and module eigengenes were correlated with radscore values.

#### Hub gene identification and risk model construction

Protein–protein interaction networks were constructed for significant modules using STRING (interaction score >0.15). Hub genes were identified using MCODE (score >3, degree >20) and MCC top-10 ranking in Cytoscape (version 3.9.0).

A multivariable Cox proportional hazards model was constructed based on hub gene expression:

riskscore=Σ(βi∗Expi)

Patients were stratified into high- and low-risk groups according to the median risk score.

### Quantification and statistical analysis

#### Statistical software and significance thresholds

All statistical analyses were performed using R software (version 4.3.3) and Python (versions 3.7 and 3.11). All tests were two-sided, and a *p*-value <0.05 (indicated by asterisks ∗ in Figure S1) was considered statistically significant unless otherwise specified.

#### Model evaluation and validation

ROC curves and AUC values were used to quantify classification performance. Sensitivity, specificity, accuracy, and F1-score were calculated at optimal cutoffs. Decision curve analysis was applied to assess net clinical benefit across a range of threshold probabilities. Calibration curves evaluated agreement between predicted and observed probabilities.

#### Survival and prognostic analyses

Overall survival differences between risk groups were assessed using Kaplan–Meier curves and log rank tests. Cox proportional hazards regression was used for univariate and multivariate analyses to identify independent prognostic factors. Model discrimination was quantified using Harrell’s C-index, which was internally validated using 1,000 bootstrap resamples.

#### Gene set enrichment and tumor microenvironment analysis

GO and KEGG enrichment analyses were performed to identify biological pathways associated with hub genes. Tumor purity, immune score, stromal score, and ESTIMATE score were calculated using the ESTIMATE algorithm and compared between risk groups.

## Supplemental information

Document S1. Figures S1–S3, Tables S1 and S2, and Method S1
