# Supplementary material for: Radiomics predicts poorly differentiated hepatocellular carcinoma and uncovers ribosomal-immune dysregulation mechanism
Source: iScience. 2026 Jan 19;29(2):114699. doi: 10.1016/j.isci.2026.114699 (PMC12925126; doi:10.1016/j.isci.2026.114699)
Supplement: Document S1. Figures S1–S3, Tables S1 and S2, and Method S1 [file mmc1.pdf]

iScience, Volume *29*

Supplemental information

Radiomics predicts poorly differentiated hepatocellular carcinoma and uncovers ribosomal-immune dysregulation mechanism

Yiping Gao, Dong Liu, Yifan Miao, Zhiqian Lou, Ziwei Luo, Yonggang Li, Hongfa Cai, Yan Zhu, and Shuangqing Chen

Method S1. MRI acquisition parameters and scanner settings, related to STAR Methods.

Table S1. Overview of radiomic feature categories and descriptions across all VOI combinations, related to table 2.

Table S2. VOI-specific radiomic features and their definitions, related to table 2.

Figure S1. Hub genes expression in HCC tumors compared to normal tissues, related to figure 4. Most of hub genes were significantly upregulated in HCC tumors compared to normal tissues in the TCGA-LIHC cohort ($log2FC > 0$, $p < 0.05$).

Figure S2. Single-VOI ablation experiments, related to discussion section. Model performance metrics of AUC, DCA and calibration curve based on single VOI of (A) Body-only, (B) Edge-only, and (C) Out-only.

Figure S3. Comparison of the performance metrics, permutation importance, SHAP summary plots for RF and the XGBoost model, and Gini importance for RF, related to discussion section. (A) Both models achieve comparable performance (AUC ~0.86–0.90) in Cross-validated and hold-out test. (B-D) RF offers more stable importance rankings, whereas XGBoost provides finer granularity in directionality at the cost of higher variance.

Method S1. MRI acquisition parameters and scanner settings (page 1-4), related to STAR M ethods.

Details of centers

The multicenter study was conducted jointly by 3 centers:

Center I: The Affiliated Suzhou Hospital of Nanjing Medical University, Suzhou, China (1.5/3.0 T, Aera/ Skyra, Siemens, Erlangen, Germany; 1.5 T, Achieva, Philip, Netherlands).

Center II: The First Affiliated Hospital of Soochow University, Suzhou, China (3.0 T, Skyra, Siemens, Erlangen, Germany; 3.0 T, Ingenia, Philip, Netherlands; 3.0 T, Signa, GE, Milwaukee WI, USA).

Center III: Third Affiliated Hospital of Naval Medical University, NO.225 Changhai Road, Shanghai, 200082, China (1.5T, Signa, GE, Milwaukee WI, USA)

Custom settings with model training and validation

Package version: Python 3.7.0, numpy 1.19.5, Pyradiomics 3.01, R 4.3.3

Settings of PyRadiomics: "normalize": True, "normalizeScale": 100, "resampledPixelSpacing": [3, 3, 3], "interpolator": sitkNearestNeighbor, "binWidth": 25

Hyperparameters of each method:

```
logistic_params = {
        'solver': ['liblinear'],
        'penalty': ['l1', 'l2'],
        'C': np.logspace(-4, 4, 9),
        'class_weight': ['balanced', None],
        'max_iter': [100, 200, 500]
    }
    rf_params = {
        'n_estimators': [100, 200, 500],
        'max_depth': [5, 7, 10],
```

```
        'min_samples_split': [2, 10, 20],
        'min_samples_leaf': [1, 2, 5],
        'max_features': ['sqrt', 'log2'],
        'random_state': [42],
    }
    decision_tree_params = {
        'criterion': ['gini', 'entropy'],
        'splitter': ['best', 'random'],
        'max_depth': [None, 5, 10, 20],
        'min_samples_split': [2, 10, 20],
        'min_samples_leaf': [1, 2, 5],
        'max_features': ['sqrt', 'log2', None],
        'random_state': [42],
    }
    svm_params = {
        'C': np.logspace(-1, 3, 10, base=2),
        'kernel': ['linear', 'rbf'],
        'gamma': ['scale', 'auto'],
    }
    knn_params = {
        'n_neighbors': [3, 5, 7, 9],
        'weights': ['uniform', 'distance'],
        'metric': ['euclidean', 'manhattan'],
```

ComBat Harmonization for Multi-Center Radiomic Data

To mitigate inter-center variability in radiomic feature distributions while preserving bi ologically relevant signals across multicenter datasets. ComBat employs empirical Baye s framework to estimate and remove center-specific batch effects. The procedure consi

sts of:

- **Standardization**: Feature-wise z-score normalization
- **Parameter Estimation**: Center-specific location (α) and scale (β) adjustments
- **Bayesian Shrinkage**: Stabilize estimates for small sample centers
- **Data Adjustment**: Apply harmonization parameters

The scanner effect is assessed by matching the statistical distributions of the feature v alues measured in ROI *j* (sample) for each scanner *i* (batch) [1]:

$$\boldsymbol{Y}_{ij} = \boldsymbol{\alpha} + \boldsymbol{X}_{ij}\boldsymbol{\beta} + \boldsymbol{\gamma}_i + \boldsymbol{\delta}_i\boldsymbol{\varepsilon}_{ij}$$

where

α: Average value of the feature;

X: Design matrix for the covariates of interest;

B: Regression coefficients for the covariates;

γi: Additive batch effect;

δ_i: Multiplicative batch effect;

ε_{ij} Error term.

By estimating the additive and multiplicative batch effect, the corrected values are obt ained using:

$$\boldsymbol{Y}_{ij}^{Combat} = \frac{\boldsymbol{Y}_{ij} - \widehat{\boldsymbol{\alpha}} - \boldsymbol{X}_{ij}\boldsymbol{\beta} - \widehat{\boldsymbol{\gamma_i}}}{\widehat{\boldsymbol{\delta_i}}} + \widehat{\boldsymbol{\alpha}}$$

Where$\hat{\alpha}$, $\hat{\beta}$, $\widehat{\gamma_i}$ and $\widehat{\delta_i}$ are estimators of α, β, γ_i, and δ_i.

Variations in MRI scanners and field strengths can introduce differences in radiomic fe atures, analogous to batch effects in genomics. In this study, MR scanners were treate d as distinct batches, with no biological covariates considered.

[1] Orlhac F, Lecler A, Savatovski J, et al. How can we combat multicenter variability in M R radiomics? Validation of a correction procedure[J]. Eur Radiol, 2021,31(4):2272-2280.

Parameters and Settings with Manufacturers

Manufacturer	Type	Sequence	TR	TE	Magnetic	Slice Thickness	FOV
SIEMENS	Aera	GRE	3.5 - 5.0	1.3 - 2.0	1.5	5 - 10	640*640/320*320
SIEMENS	Skyra	GRE	3.0 - 4.5	1.2 - 1.8	3.0	5 - 10	320*320/640*640
GE	Signa	GRE	3.5 - 4.5	1.5 - 2.0	3.0	4 - 10	512*512
Philips	Achieva	GRE	3.5 - 5.0	1.3 - 1.9	1.5	5.7 – 10.5	480*480
GE	Signa	GRE	3.7	1.7	1.5	8	512*512

GRE: Gradient Echo; TR: repetition time; TE: echo time; SE: spin-echo; FOV: field of view

Table S1. Overview of radiomic feature categories and descriptions across all VOI combinations, related to table 2.

Category	Defination	Biological/radiological significance
First-Order Features	Global statistics based on voxel gray intensity (such as mean, variance, entropy, percentile, etc.).	Reflect the overall gray distribution characteristics of the tumor, such as heterogeneity, intensity range and metabolic activity.
GLCM (Gray-Level Co-occurrence Matrix)	Joint probability distribution of adjacent voxel gray pairs	Quantify the direction, uniformity and local gray change of texture.
GLDM (Gray-Level Dependence Matrix)	The dependence of gray value on surrounding voxels.	Capture the spatial correlation of the internal structure of the tumor.
GLSZM (Gray-Level Size Zone Matrix)	The spatial distribution of continuous regions with the same gray level.	Characterize the distribution pattern of homogeneous regions within tumors
GLRLM (Gray Level Run Length Matrix)	Quantifies gray level runs-the length in number of pixels.	Reflects the uniformity of tissue structure and tumor heterogeneity.

Table S2. VOI-specific radiomic features and their definitions, related to table 2.

Category	Specific characteristics
**Body VOI**	
First-Order Features	original_firstorder_10Percentile, lbp-2D_firstorder_InterquartileRange, lbp-2D_firstorder_Variance, lbp-3D-k_firstorder_Kurtosis, wavelet-LLH_firstorder_MeanAbsoluteDeviation, wavelet-LLH_firstorder_Range, wavelet-LHL_firstorder_InterquartileRange, wavelet-LHL_firstorder_RootMeanSquared, wavelet-HLH_firstorder_Maximum, wavelet-HHL_firstorder_10Percentile, wavelet-LLL_firstorder_10Percentile
Texture Features	
GLSZM	original_glszm_SizeZoneNonUniformityNormalized, lbp-3D-k_glszm_SmallAreaHighGrayLevelEmphasis, logarithm_glszm_SizeZoneNonUniformityNormalized, squareroot_glszm_SizeZoneNonUniformityNormalized, wavelet-LLH_glszm_SmallAreaEmphasis, wavelet-LLH_glszm_SmallAreaLowGrayLevelEmphasis, wavelet-LHL_glszm_LowGrayLevelZoneEmphasis, wavelet-LHH_glszm_SizeZoneNonUniformityNormalized, wavelet-LHH_glszm_SmallAreaHighGrayLevelEmphasis, wavelet-LHH_glsz

	m_ZoneEntropy, wavelet-HLL_glszm_GrayLevelNonUniformityNormalized, wavelet-HLH_glszm_SmallAreaLowGrayLevelEmphasis
GLRLM	lbp-3D-m1_glrlm_RunLengthNonUniformityNormalized, wavelet-LLH_glrlm_ShortRunHighGrayLevelEmphasis, wavelet-HLL_glrlm_LongRunHighGrayLevelEmphasis, wavelet-HHL_glrlm_LongRunLowGrayLevelEmphasis
GLDM	wavelet-LLH_gldm_LargeDependenceLowGrayLevelEmphasis
GLCM	lbp-3D-k_glcm_ClusterProminence, wavelet-LLH_glcm_Contrast, wavelet-LLH_glcm_Correlation, wavelet-LLH_glcm_DifferenceAverage, wavelet-LLH_glcm_Id, wavelet-LLH_glcm_Idm, wavelet-LLH_glcm_Idn, wavelet-LLH_glcm_InverseVariance, wavelet-LHH_glcm_Imc2, wavelet-HLH_glcm_Imc2
**Out VOI**	
First-Order Features	original_firstorder_10Percentile, gradient_firstorder_10Percentile, lbp-3D-m1_firstorder_InterquartileRange, square_firstorder_InterquartileRange, wavelet-LLH_firstorder_Maximum, wavelet-LHL_firstorder_10Percentile, wavelet-LHL_firstorder_RootMeanSquared, wavelet-HLH_firstorder_Maximum, wavelet-HLH_firstorder_Range, wavelet-HHL_firstorder_10Percentile
Texture Features	
GLSZM	original_glszm_ZoneEntropy, lbp-3D-k_glszm_GrayLevelNonUniformityNormalized, lbp-3D-k_glszm_GrayLevelVariance, logarithm_glszm_SizeZoneNonUniformityNormalized, logarithm_glszm_ZoneEntropy, squareroot_glszm_SizeZoneNonUniformityNormalized, squareroot_glszm_ZoneEntropy, wavelet-LLH_glszm_SmallAreaEmphasis, wavelet-LHH_glszm_SmallAreaHighGrayLevelEmphasis
GLRLM	wavelet-LLH_glrlm_ShortRunHighGrayLevelEmphasis
GLDM	wavelet-LLH_gldm_LargeDependenceLowGrayLevelEmphasis
GLCM	wavelet-LHL_glcm_ClusterShade, wavelet-HLL_glcm_ClusterShade
NGTDM	Feature<br>lbp-3D-k_ngtdm_Complexity
**Edge VOI**	
First-Order Features	gradient_firstorder_10Percentile, lbp-3D-m1_firstorder_Variance, lbp-3D-k_firstorder_Uniformity, wavelet-LLH_firstorder_Maximum, wavelet-LHL_firstorder_InterquartileRange, wavelet-HHL_firstorder_10Percentile, wavelet-HHL_firstorder_RootMeanSquared, wavelet-LLL_firstorder_Skewness
Texture Features	
GLSZM	lbp-3D-k_glszm_SmallAreaEmphasis, lbp-3D-k_glszm_SmallAreaHighGrayLevelEmphasis, logarithm_glszm_SizeZoneNonUniformityNormalized, squareroot_glszm_SizeZoneNonUniformityN

	ormalized, wavelet-HHL_glszm_SmallAreaEmphasis, wavelet-LLL_glszm_ZoneEntropy
GLRLM	wavelet-LLH_glrlm_ShortRunHighGrayLevelEmphasis
GLDM	lbp-3D-k_gldm_GrayLevelVariance, wavelet-LLH_gldm_LargeDependenceLowGrayLevelEmphasis, wavelet-LHL_gldm_LargeDependenceHighGrayLevelEmphasis
**Body+Edge VOI**	
First-Order Features	lbp-3D-k_firstorder_Maximum, square_firstorder_RootMeanSquared, wavelet-LLH_firstorder_Range, wavelet-LHL_firstorder_InterquartileRange, wavelet-HHL_firstorder_10Percentile, wavelet-HHH_firstorder_RootMeanSquared
Texture Features	
GLSZM	lbp-3D-k_glszm_GrayLevelVariance, wavelet-HLL_glszm_LowGrayLevelZoneEmphasis
GLRLM	wavelet-LLH_glrlm_ShortRunEmphasis,
GLDM	original_gldm_LargeDependenceLowGrayLevelEmphasis, logarithm_gldm_LargeDependenceLowGrayLevelEmphasis, squareroot_gldm_LargeDependenceLowGrayLevelEmphasis
GLCM	wavelet-LLH_glcm_MCC, wavelet-HLH_glcm_MCC
**Body+Out VOI**	
First-Order Features	lbp-2D_firstorder_Entropy, wavelet-LLH_firstorder_Range, LHL_firstorder_MeanAbsoluteDeviation, wavelet-HLH_firstorder_RootMeanSquared, wavelet-HHL_firstorder_10Percentile
Texture Features	
GLSZM	wavelet-HLL_glszm_LowGrayLevelZoneEmphasis
GLRLM	wavelet-LLH_glrlm_RunLengthNonUniformityNormalized
GLCM	exponential_glcm_JointEntropy, exponential_glcm_SumEntropy, gradient_glcm_DifferenceEntropy, gradient_glcm_JointEntropy, gradient_glcm_SumEntropy, lbp-2D_glcm_DifferenceEntropy, lbp-2D_glcm_JointEntropy, lbp-2D_glcm_SumEntropy, lbp-3D-m1_glcm_JointEntropy, lbp-3D-m1_glcm_SumEntropy, lbp-3D-m2_glcm_DifferenceEntropy, lbp-3D-m2_glcm_SumEntropy, square_glcm_JointEntropy, square_glcm_SumEntropy, wavelet-HLH_glcm_MCC
**Edge+Out VOI**	
First-Order Features	lbp-2D_firstorder_Entropy, lbp-3D-m1_firstorder_Entropy, lbp-3D-k_firstorder_Maximum, square_firstorder_Median, wavelet-LHL_firstorder_InterquartileRange

Texture Features	
GLSZM	lbp-3D-k_glszm_SmallAreaHighGrayLevelEmphasis, wavelet-LHH_glszm_SmallAreaEmphasis, wavelet-HLL_glszm_LowGrayLevelZoneEmphasis
GLRLM	wavelet-LLH_glrlm_RunEntropy, wavelet-LLH_glrlm_RunLengthNonUniformityNormalized, wavelet-LLH_glrlm_ShortRunEmphasis
GLDM	original_gldm_LargeDependenceLowGrayLevelEmphasis, logarithm_gldm_LargeDependenceLowGrayLevelEmphasis, squareroot_gldm_LargeDependenceLowGrayLevelEmphasis, wavelet-HHH_gldm_DependenceVariance
GLCM	exponential_glcm_DifferenceEntropy, exponential_glcm_JointEntropy, exponential_glcm_SumEntropy, gradient_glcm_DifferenceEntropy, gradient_glcm_JointEntropy, gradient_glcm_SumEntropy, lbp-2D_glcm_JointEntropy, lbp-2D_glcm_SumEntropy, lbp-3D-m1_glcm_SumEntropy, lbp-3D-m2_glcm_JointEntropy, lbp-3D-m2_glcm_SumEntropy, square_glcm_DifferenceEntropy, square_glcm_JointEntropy, wavelet-HLH_glcm_MCC, wavelet-HHL_glcm_Autocorrelation, wavelet-LLH_glcm_MCC
**Integrated VOI**	
First-Order Features	logarithm_firstorder_Minimum, wavelet-LLH_firstorder_RootMeanSquared, wavelet-LHL_firstorder_MeanAbsoluteDeviation, wavelet-HHH_firstorder_RobustMeanAbsoluteDeviation, wavelet-LLL_firstorder_InterquartileRange
Texture Features	
GLSZM	lbp-3D-k_glszm_GrayLevelVariance, wavelet-LLH_glszm_ZonePercentage
GLRLM	logarithm_glrlm_ShortRunHighGrayLevelEmphasis, squareroot_glrlm_ShortRunHighGrayLevelEmphasis
GLCM	wavelet-LLH_glcm_Autocorrelation, wavelet-HHL_glcm_JointEntropy
GLDM	wavelet-LLH_gldm_SmallDependenceEmphasis

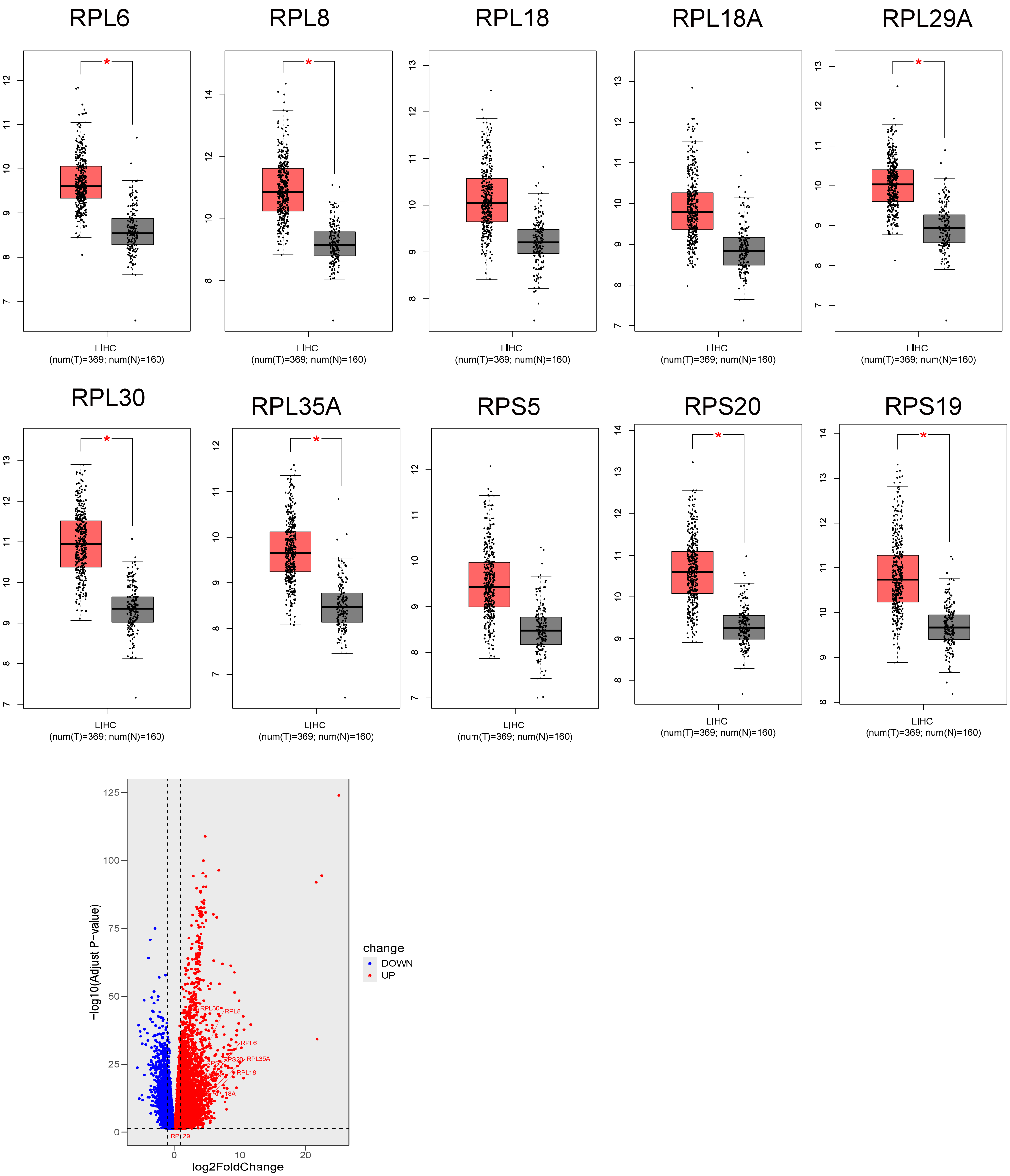

Figure S1. Hub genes expression in HCC tumors compared to normal tissues, related to Figure 4. Most of hub genes were significantly upregulated in HCC tumors compared to normal tissues in the TCGA-LIHC cohort (log2FC > 0, p < 0.05; Figure S1). Statistical significance was defined as p < 0.05 (*), p < 0.01 (**), and p < 0.001 (***).

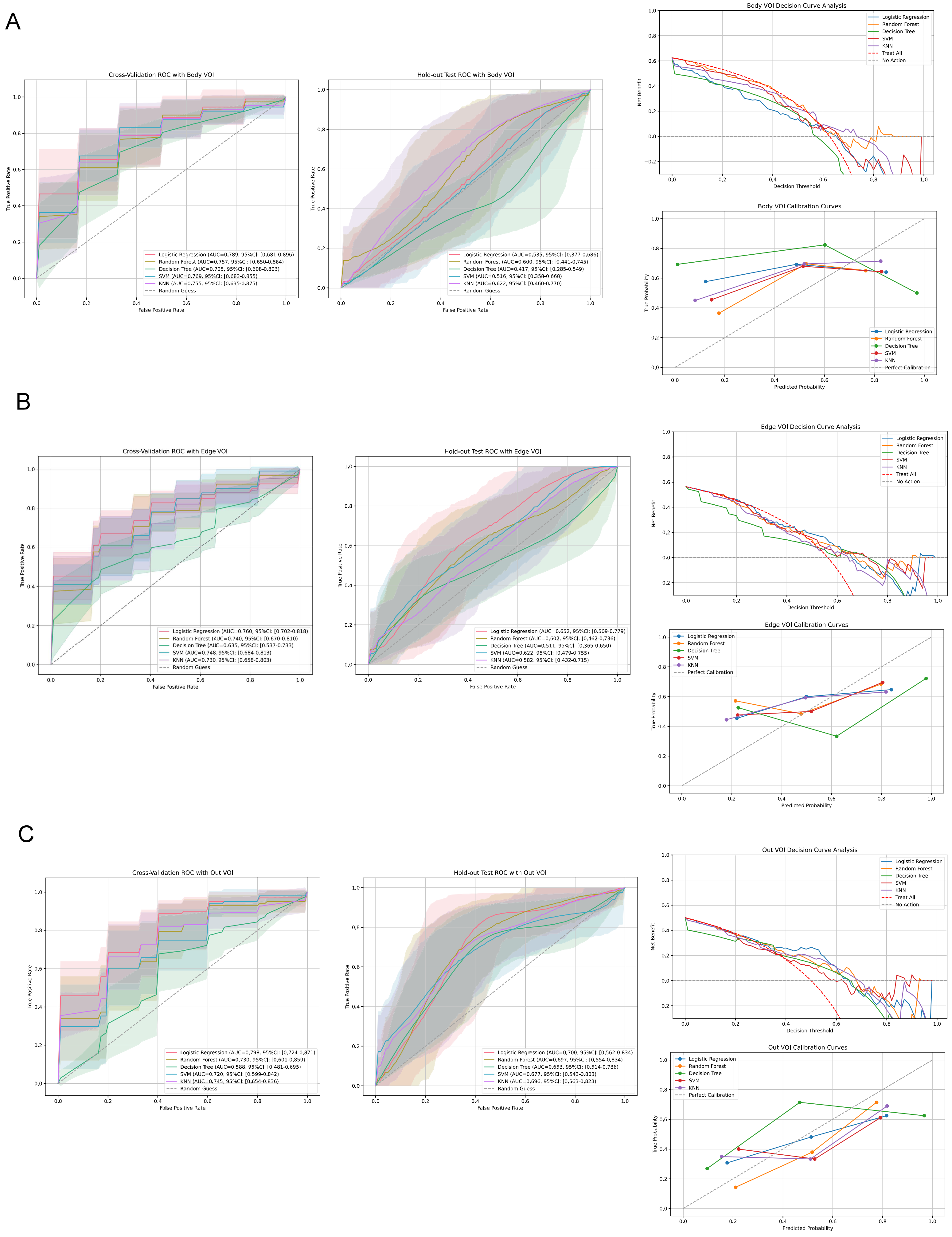

Figure S2 Single-VOI ablation experiments, related to discussion section. Model performance metrics of AUC, DCA and calibration curve based on single VOI of (A) Body-only, (B) Edge-only, and (C) Out-only.

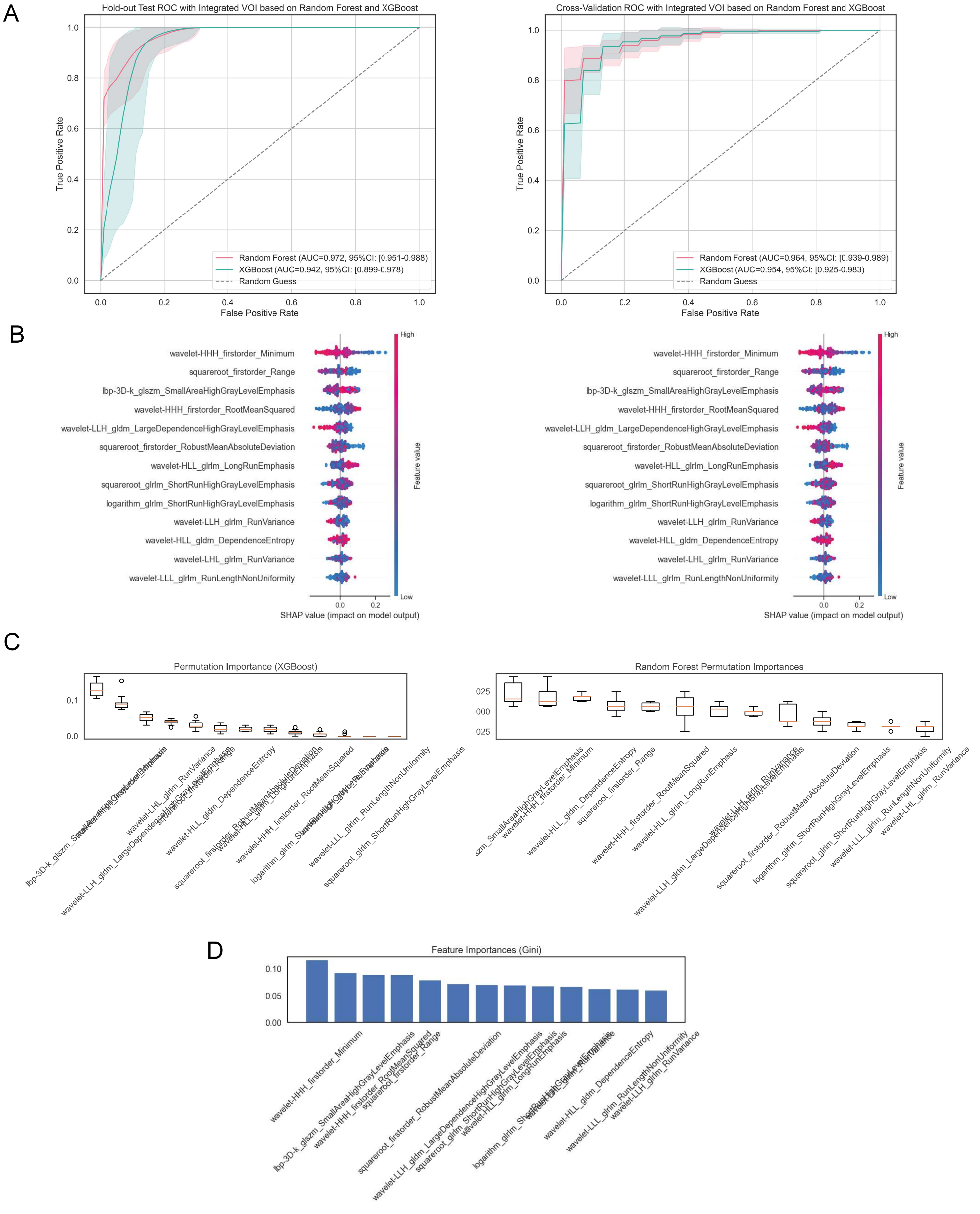

Figure S3. Comparison of the AUC, shap plots, and permutation importance for RF and the XGBoost model, and Gini importance for RF model, related to discussion section. (A) Both models achieve comparable performance (AUC ~0.86–0.90) in Cross-validated and hold-out test.(B-D) RF offers more stable importance rankings, whereas XGBoost provides finer granularity in directionality at the cost of higher variance.
